# A high throughput generative vector autoregression model for stochastic synapses

**DOI:** 10.3389/fnins.2022.941753

**Published:** 2022-08-18

**Authors:** Tyler Hennen, Alexander Elias, Jean-François Nodin, Gabriel Molas, Rainer Waser, Dirk J. Wouters, Daniel Bedau

**Affiliations:** ^1^Institut für Werkstoffe der Elektrotechnik 2 (IWE II), RWTH Aachen University, Aachen, Germany; ^2^Western Digital San Jose Research Center, San Jose, CA, United States; ^3^CEA, LETI, Grenoble, France; ^4^Weebit Nano Ltd., Grenoble, France

**Keywords:** neuromorphic computing, machine learning, time series, emerging technologies, stochastic model, ReRAM, neural networks, nanotechnology

## Abstract

By imitating the synaptic connectivity and plasticity of the brain, emerging electronic nanodevices offer new opportunities as the building blocks of neuromorphic systems. One challenge for large-scale simulations of computational architectures based on emerging devices is to accurately capture device response, hysteresis, noise, and the covariance structure in the temporal domain as well as between the different device parameters. We address this challenge with a high throughput generative model for synaptic arrays that is based on a recently available type of electrical measurement data for resistive memory cells. We map this real-world data onto a vector autoregressive stochastic process to accurately reproduce the device parameters and their cross-correlation structure. While closely matching the measured data, our model is still very fast; we provide parallelized implementations for both CPUs and GPUs and demonstrate array sizes above one billion cells and throughputs exceeding one hundred million weight updates per second, above the pixel rate of a 30 frames/s 4K video stream.

## Introduction

Recent trends in computing hardware have placed increasing emphasis on neuromorphic architectures implementing machine learning (ML) algorithms directly in hardware. Such bio-inspired approaches, through in-memory computation and massive parallelism, excel in new classes of computational problems and offer promising advantages with respect to power consumption and error resiliency. While CMOS-based neuromorphic computing (NC) implementations have made substantial progress recently, new materials and physical mechanisms may ultimately provide better opportunities for energy efficiency and scaling (Burr et al., 2017; Milo et al., 2020; Sangwan and Hersam, 2020).

**Figure 1:**
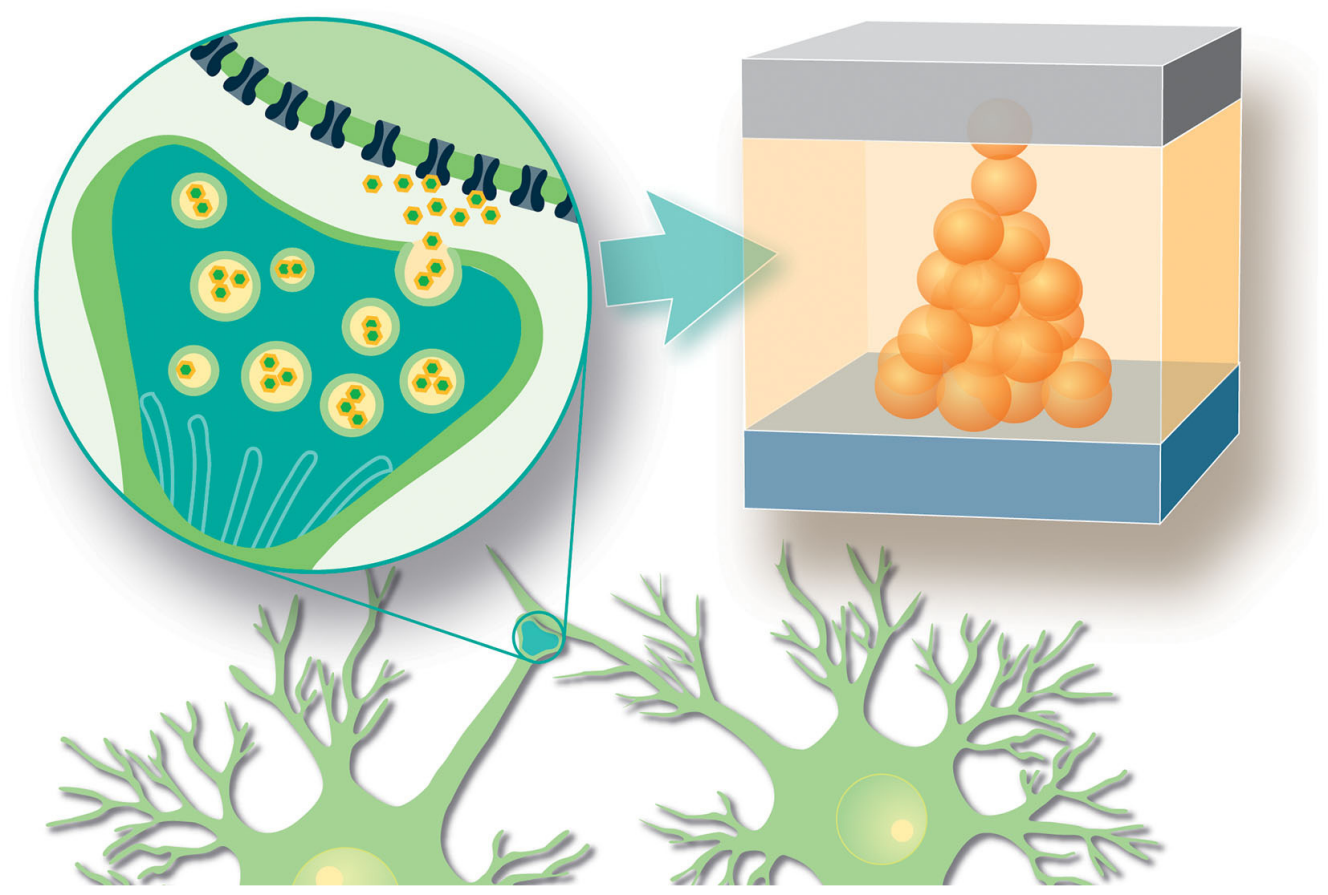
In analogy to biological synapses, two terminal solid state nanodevices such as ReRAM can store synaptic weights as electrical resistance states. The devices, consisting simply of patterned metal-insulator-metal material stacks, have an adjustable resistance level determined by the ionic configuration inside the insulating layer. This nano-ionic mechanism also exhibits non-ideal properties such as stochasticity and noise.

A specific functionality required in NC applications is the ability to mimic synaptic connections and plasticity by allowing the storage of large numbers of interconnected and continuously adaptable resistance values. Several candidate memory technologies such as MRAM, ReRAM, PCM, CeRAM, are emerging to cover this behavior using different physical mechanisms (Chen et al., 2014; Liu et al., 2014; You Zhou and Ramanathan, 2015; Yu and Chen, 2016). Among these, ReRAM is attractive for its simplicity of materials and device structure, providing the necessary CMOS compatibility and scalability (Waser et al., 2009). ReRAM is essentially a two terminal nanoscale electrochemical cell, whose variable resistance state is based on manipulation of the point defect configuration in the oxide material (depicted in Figure 1). This redox-based switching mechanism is intrinsically analog, allowing stable resistance levels to be stored and adjusted through application of bipolar voltage stimuli. However, non-idealities such as stochasticity, nonlinearity, and noise are prominent features of these devices that critically impact the performance of neuromorphic systems composed of them (Kim et al., 2018).

Modern ML models have reached an astonishingly large and ever-increasing size, with recent examples exceeding a hundred billion weights (Brown et al., 2020). Before comparable neuromorphic hardware using artificial solid-state synapses can become a reality, large-scale network designs need to first be implemented and evaluated in computer simulations. Training, validation, and optimization of such networks is a process that involves a huge number of simulated devices, voltage pulses, and current readouts. Within this process, it is important to accurately consider the constraints of the underlying hardware in detail. Therefore, lightweight, fast, and accurate stochastic simulations of the individual synaptic devices is a key requirement.

Traditionally, device modeling begins with a physical description of the materials and processes involved. In the case of ReRAM, the physical situation is immensely complicated with many degrees of freedom, and accurate modeling is a wide-scale and ongoing research undertaking. Efforts in this direction are motivated by advancing an understanding of physical and chemical dependencies that can in principle inform design choices on physically justified grounds. In the past decade, many different computational techniques have been employed to furnish device models, from ab initio density-functional theory (DFT), molecular dynamics (MD), kinetic Monte Carlo (KMC), finite element method (FEM), as well as ordinary differential equation (ODE) and differential algebraic equation (DAE) solvers (Ascoli et al., 2015; Jiang and Stewart, 2017; Messaris et al., 2018; Stewart, 2019; Kopperberg et al., 2021). The resulting models exist on a spectrum of physical abstraction, such that the cost of increasing computational speed is generally a trade-off in physical accuracy/detail (Ielmini and Milo, 2017).

Device models that naturally encompass stochasticity do so at the cost of complexity needed to compute the physical scenario in high detail. For example, atomistic KMC simulates switching processes with atomic precision and is inherently stochastic but requires hours of computation per cycle even for small individual cell volumes (e.g., 125 nm^2^ Abbaspour et al., 2020). At the other end of the spectrum, dynamic models based on numerical solutions of ODE systems are designed to run significantly faster while sometimes aiming to remain physically realistic. However, their higher speed invariably comes at the cost of approximations, simplifications, and omissions of physical reality. Typically, device operation is distilled to a dynamical description of one or two state variables, such as a conducting filament length, radius, or a defect concentration.

Due in part to ambiguity in their high dimensional parameter space, a given ODE model encompasses a diverse range of possible cell behaviors and has the flexibility to approximately match measurement data (Mayer et al., 2010; Reuben et al., 2019). However, fitting the model to data is commonly an *ad-hoc*, manual, and/or unspecified procedure. Having dispensed with the atomistic sources of variability, ODE models are fully deterministic by default. Where stochasticity is required, it is accounted for by injecting noise into the state variables or parameters of the model (Maria Puglisi et al., 2015; Li et al., 2017; Bengel et al., 2020). Due to the unique experimental challenges posed by electrical measurement of ReRAM, the data used for fitting is not necessarily statistically sufficient nor measured under relevant electrical conditions and timescales. While models can be tuned by hand to roughly match the dispersion observed in a measurement (Chen and Yu, 2015; Jiang et al., 2016), they generally fail to accurately reproduce the complex statistical properties of actual devices.

The main purpose of ODE device models is to be computationally efficient enough to support circuit simulation. Still, nonlinear ODE solvers require many finely spaced timesteps and a considerable amount of total time to compute dynamical trajectories. Although they have been successfully used to demonstrate small scale circuitry such as logic elements and small crossbar arrays (Bocquet et al., 2014; Huang et al., 2017; Siemon et al., 2019; Wald and Kvatinsky, 2019), benchmarks or indications of run time for ODE-based simulations have so far not been supplied. Except for extremely small ML model sizes on the order of 10^3^ weights or below, demonstrations of network performance are expected to remain computationally intractable *via* conventional circuit simulation.

In this article, we address these device modeling challenges with a new type of generative model for arrays of artificial synapses. The main objective of the model is to accurately reproduce the statistical properties of fabricated devices while remaining computationally lightweight. Starting with newly available electrical measurement data as an input, this phenomenological model is systematically fit using a well defined statistical regression analysis. The exclusive use of easily computable analytical expressions provides close quantitative agreement with relevant experimental observation. Taking advantage of parallel resources on a modern CPU and GPU, we demonstrate the ability to simulate hundreds of millions of synaptic connections with over 10^8^ weight updates per second. With its high throughput and low memory footprint, the model can be usefully employed to simulate large arrays of solid-state synapses for investigation of emerging NC concepts on a large scale.

## Methods

The basic requirement for an electronic device serving as an artificial synapse is to moderate the flow of electrical signals through connections in a network. Left undisturbed, the device ideally maintains a fixed weight, or dependence between the voltage across the two device terminals, *U*, and the resulting current through the device, *I*. Further, for learning there must be some means of affecting the weight in a durable way. ReRAMs are bipolar devices that have an adjustable (potentially nonlinear) non-volatile resistance state, which is based on the size and shape of a conducting filament that partially or fully bridges the insulating gap of the oxide material. Simplistically, when *U* exceeds certain threshold levels, the resistance state begins to transition toward lower or higher values depending on the voltage polarity, which corresponds to growth and shrinkage of the conducting filament. When the filament only partially bridges the insulating gap, conduction may be limited for example by tunneling through a Schottky barrier of a material interface, leading to a relatively high resistance levels (Yang et al., 2008; Waser et al., 2009). As the filament grows and gradually bridges the gap, the resistance decreases as conduction transitions into the ohmic type.

In designing our model, we place high priority on speed and fitting accuracy. One of the beginning assumptions is that in every possible device state, the current can be represented by a linear mixture of two fixed polynomials in *U*. These two polynomials, which are each estimated from a fit to measurement data, can be thought of as limiting cases for the highest possible high resistance state, *I*HHRS(*U*), and lowest possible low resistance state, *I*LLRS(*U*). The device current in all possible resistance states is then given by


(1) $$\begin{array}{l} I(r,U)=rI_{\text{HHRS}}(U)+(1-r)I_{\text{LLRS}}(U), \end{array}$$

conveniently reducing the description of the conduction in the material to a single state variable 0 < *r* < 1. This set of functions can be efficiently evaluated by Horner's algorithm and serve as a close enough approximation to the true non-linear conduction behavior for our purposes.

**Figure 2:**
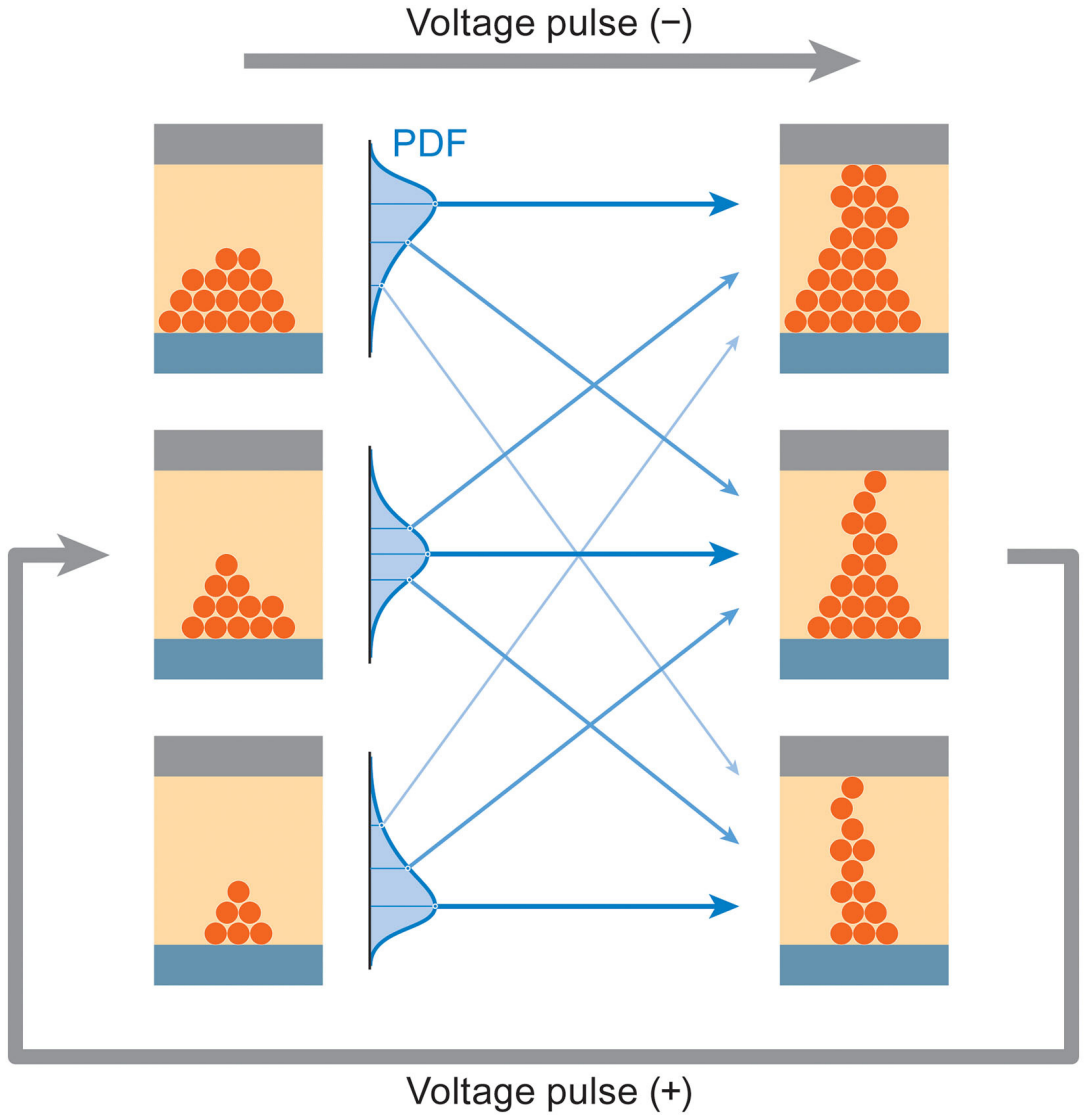
Resistance states reached in a synaptic ReRAM device through application of voltage pulses exhibit a probabilistic dependence on past states, leading to long-range correlations that also involve other parameters such as the voltage thresholds required for switching. Starting with effectively infinite state possibilities, represented by the three cells on the left, an applied voltage pulse brings about a set of transition probabilities to many possible future states (right).

In ReRAM, the overall resistance state as well as the transition behavior is affected by a vast number of different possible configurations of ionic defects in the material, giving rise to the observed stochastic behavior and history dependence (Figure 2). Rather than attempting to describe the ionic transport physically, we turn instead to measurement data to directly provide the necessary statistical information. A discrete multivariate stochastic process based on a Structural Vector Autoregression (SVAR) model is fit to the data and used to generate latent variables that guide the state evolution of simulated memory cells. As a cell is exposed to voltage signals, new terms of the SVAR model are realized by a sum of easily computable linear transformations of past states and pseudorandom vectors.

As an overview, the experimental and simulation approach that will be elaborated in this section can be shortly summarized as follows:

1. A fabricated ReRAM cell is experimentally driven through a large number of resistance cycles by applying a continuous periodic voltage signal while measuring the resulting current.
2. A time series of feature vectors, ***x***_*n*_, composed of resistance values and switching threshold voltages, is extracted from each of the measured cycles.
3. A discrete stochastic process, $x_{n}^{*}$, is constructed to enable generation of simulated feature vectors that reproduce the measured distributions as well as the long-range correlation structure of ***x***_*n*_.
4. An array of simulated cells are instantiated according to independent realizations of $x_{n}^{*}$ to represent cycle-to-cycle variations, together with a random scaling vector ***s***_*m*_ to represent device-to-device variations.
5. Two programming methods are exposed for each cell; one to apply voltages and another to make realistic current readouts. Applied voltages above the generated thresholds alter the device state, following an empirical structure which encodes the resistance transition behavior and allows access to a range of resistance states. Each voltage driven resistance cycle triggers the generation of new stochastic terms from $x_{n}^{*}$, which govern the progression to future states.

### Data collection

For the purposes of stochastic modeling, electrical measurement data is needed that capture relevant information about the internal state of a memory cell and its variation cycle-to-cycle (CtC) and device-to-device (DtD). However, ReRAM measurements performed at operational speed typically make exclusive use of rectangular voltage pulse sequences, which yield very little useful state information. On the other hand, measurements applying continuously swept voltage signals while sampling the resulting current are more suitable because much more information is collected each cycle, such as switching threshold voltages, current-voltage nonlinearity, resistance states, and transition behavior.

Conventionally, measurements employing voltage sweeps are carried out using the source measure units (SMUs) of commercial semiconductor parameter analyzers (SPAs). However, SMUs make heavy use of averaging to measure noisy signals at high resolution and thus sample too slowly to collect cycling data in a meaningful quantity. Furthermore, because two-terminal switching devices are prone to electrical instability and runaway transitions, voltage sweeping measurements usually require integrated current limiting transistors to avoid destruction or rapid degradation of the cell. This presents a significant fabrication overhead and limits the materials available for study. In light of these challenges, the input data for the present stochastic model was acquired using a custom measurement technique, introduced in detail in a recent publication (Hennen et al., 2021). The setup uses an external current-limiting amplifier circuit to allow for collection of sweeping measurements at over six orders of magnitude higher speeds than SMUs, while also eliminating the cumbersome requirement of on-chip current limiting.

**Figure 3:**
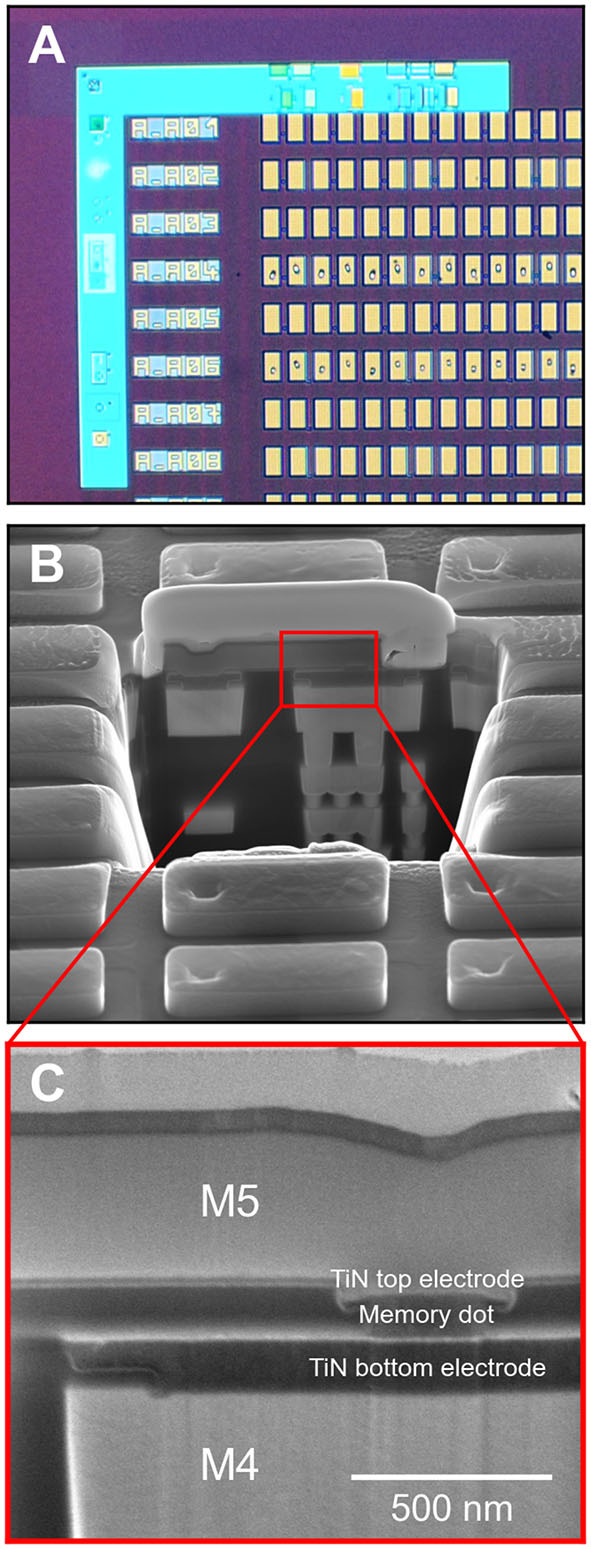
Scanning electron micrographs of the ReRAM cell design used for electrical measurement. **(A)** shows an optical image of the array of contact pads, **(B)** shows a cross-section of a cell, and **(C)** shows a zoom-in of the resistive memory between metalization layers M4 and M5.

The ReRAM cell used for measurement of cycling statistics was integrated in the back end of line of a 130 nm CMOS process, between M4 and M5 aluminum metal lines (Figure 3). On M4, a damascene TiN *via* followed by a patterned TiN bottom electrode were processed, forming the inert electrode of the device. The memory stack was then deposited. First, 10 nm HfO_2_ deposited by atomic layer deposition (using HfCl_4_ and H_2_O precursors) acts as the resistive switching layer (Nail et al., 2016). Then, a 20 nm Ti scavenging layer was deposited by physical vapor deposition, allowing creation of oxygen vacancies within the HfO_2_ during the memory operation. A 100 nm TiN top layer was used to cap the device. Deep ultraviolet photolithography and dry etching were used to pattern the memory dot, defining the active area. A SiN capping layer was used to isolate the memory from adjacent cells. Top vias were then opened by photolithography and dry etching in order to contact the memory dots. Finally, aluminum M5 was deposited and patterned to complete the process flow.

**Figure 4:**
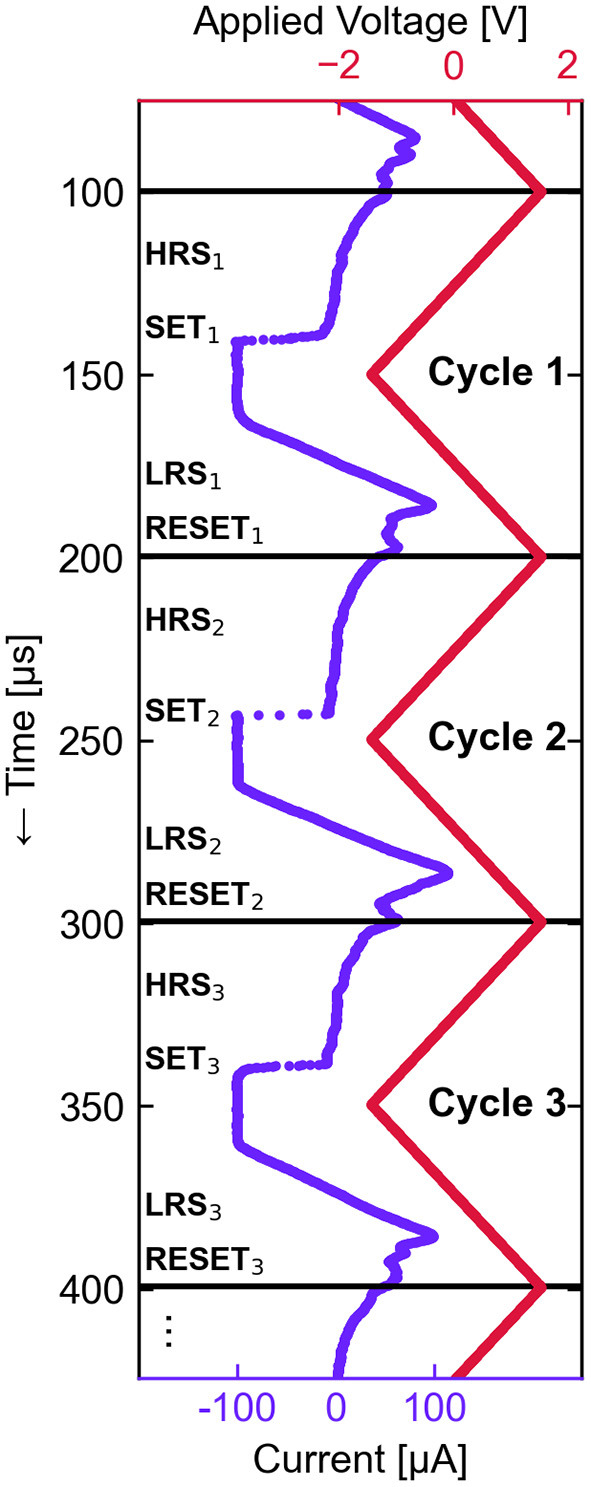
Measured time dependence of *I* and *U* waveforms resulting from the ReRAM cycling experiment. The waveforms are divided into 10^6^ indexed cycles, the first three of which are shown. From this dataset, the periodic temporal sequence of the states and events of each cycle (HRS_*n*_, SET_*n*_, LRS_*n*_, RESET_*n*_) is extracted and subject to statistical modeling.

The measured device was electrically isolated with contact pads leading directly to the top and bottom device electrodes, with no access transistor or added series resistance. Using a fixed 100 μA current limit in the SET polarity, the pristine cell was electroformed by application of 100 μs duration triangular pulses with incrementally increasing amplitude until a current jump was recorded near 3 V. For all subsequent cycling, a 1.5 V amplitude 10 kHz triangular waveform was applied. The cell was first exercised for 2.4 × 10^6^ cycles before 10^6^ additional cycles were collected for analysis. Current (*I*) and voltage (*U*) waveforms were simultaneously recorded with 8-bit resolution and with a sample rate of 1,042 samples per cycle. The measured current array was smoothed with a moving average filter to improve the quality of the raw data before further analysis. An adaptive rectangular window size was used to preserve current steps in the signal, with the maximum window size of 25 samples gradually reducing to a minimum of 3 samples at the pre-detected locations of SET transitions of each cycle. After smoothing, the contiguous *I* and *U* waveforms were split into indexable cycles at most positive value of the periodic applied voltage (see Figure 4).

**Figure 5:**
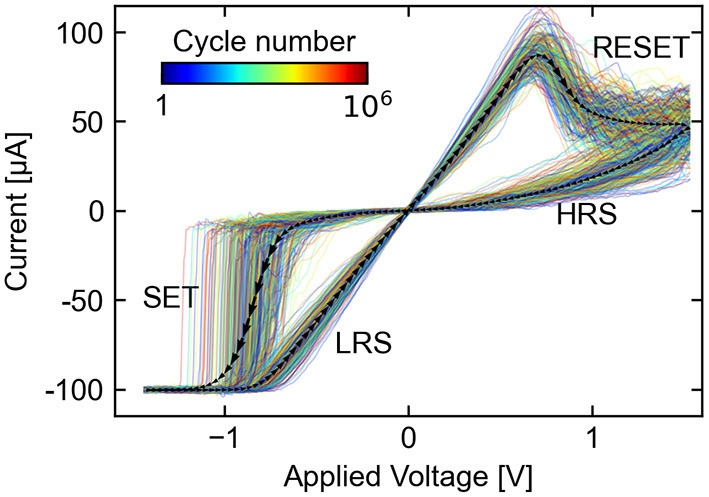
A subset of the 10^6^ measured (*I, U*) cycles used as input to the stochastic model. The black arrowed path shows the average (*I, U*) curve and its temporal direction. Different cycle indices are represented by colored paths, which show significant statistical variation.

Each cycle exhibits the following temporal sequence of states and events: a high resistance state (HRS), a transition (SET) out of the HRS into the following low resistance state (LRS), and finally another transition (RESET) into the next HRS. Current vs. voltage (*I, U*) plots for a subset of the collected cycles are shown in Figure 5, which highlights the significant stochastic CtC variations. The observed characteristics are typical for ReRAM devices subjected to voltage-controlled sweeps — on average, there is relatively higher voltage non-linearity in the HRS than in the LRS, and a large proportion of the SET transitions are abrupt with respect to the applied voltage. The SET transition times as defined by the time spent between –30 μA and –90 μA is connected to the voltage sweep rate, and was distributed between 100 ns and 5 μs in this case. The RESET transitions, in contrast, proceed relatively gradually over a voltage range of approximately 700 mV, following a concave transition curve with N-type negative differential resistance.

### Feature extraction

The full *I, U* cycling measurement just described consists of over 16 GB of numerical data and would not be practical to model on a point-by-point basis. Therefore, we aim to compress the dataset while retaining enough information such that the full (*I, U*) characteristics can be approximately reconstructed from the compressed representation. Accordingly, the full dataset is reduced to a vector time series of distinguishing features of each cycle. Four scalar features were chosen for extraction: the value of the HRS, *R*_*H*_[Ω], the SET threshold voltage, *U*_*S*_[*V*], the value of the LRS, *R*_*L*_[Ω], and the RESET voltage, *U*_*R*_[*V*]. We denote the series as


(2) $$\begin{array}{l} x_{n}=\left[\begin{array}{c} R_{H,n}\\ U_{S,n}\\ R_{L,n}\\ U_{R,n} \end{array}\right]={\left[\begin{array}{c} R_{H}\\ U_{S}\\ R_{L}\\ U_{R} \end{array}\right]}_{n}, \end{array}$$

where *n* = {1, 2, …, 10^6^} is the set of cycle indices. The feature vector elements, whose precise definition follows, are chronologically ordered from top to bottom as they occur in the measurement dataset.

The SET voltage *U*_*S*_, or the voltage where the cell resistance abruptly decreases, is extracted from each cycle as the absolute value of the linearly interpolated *U* corresponding to the first level crossing of *I* = −50 μA. The RESET voltage *U*_*R*_, defined as the voltage where the reset process begins, is determined from the *I* datapoints by peak detection using simple comparison of neighboring samples. Here, only the increasing section of the voltage sweep with *U* > 0 is considered. The voltage corresponding to the first encountered peak with prominence ≥5 μA is taken as the RESET voltage. If no peak satisfies this criterion, the peak with maximum prominence is taken instead.

The device current for any static state is approximated in our model as a polynomial function of the applied voltage. The values of *R*_*H*_ and *R*_*L*_ are likewise extracted from least squares polynomial fits to appropriate subsets of the measured (*I, U*) data of each cycle. The HRS is fit with a 5th degree polynomial on the decreasing *U* sweep in the variable range *U*_*S*_+0.1 V ≤ *U* ≤ 1.5 V and −25 μA ≤ *I* ≤ 80 μA, and the LRS is fit with a 3rd degree polynomial on the increasing part of the *V* sweep in the range −0.7 V ≤ *U* ≤ *U*_*R*_−0.05 V and −80 μA ≤ *I* ≤ 120 μA. The fits are constrained such that the 0th order coefficient equals 0 A, and the 1st order coefficient is ≥1 nA/V. The values of *R*_*H*_ and *R*_*L*_ are then defined as the static resistance of the respective polynomials at a fixed voltage *U*_0_ = 200 mV.

**Figure 6:**
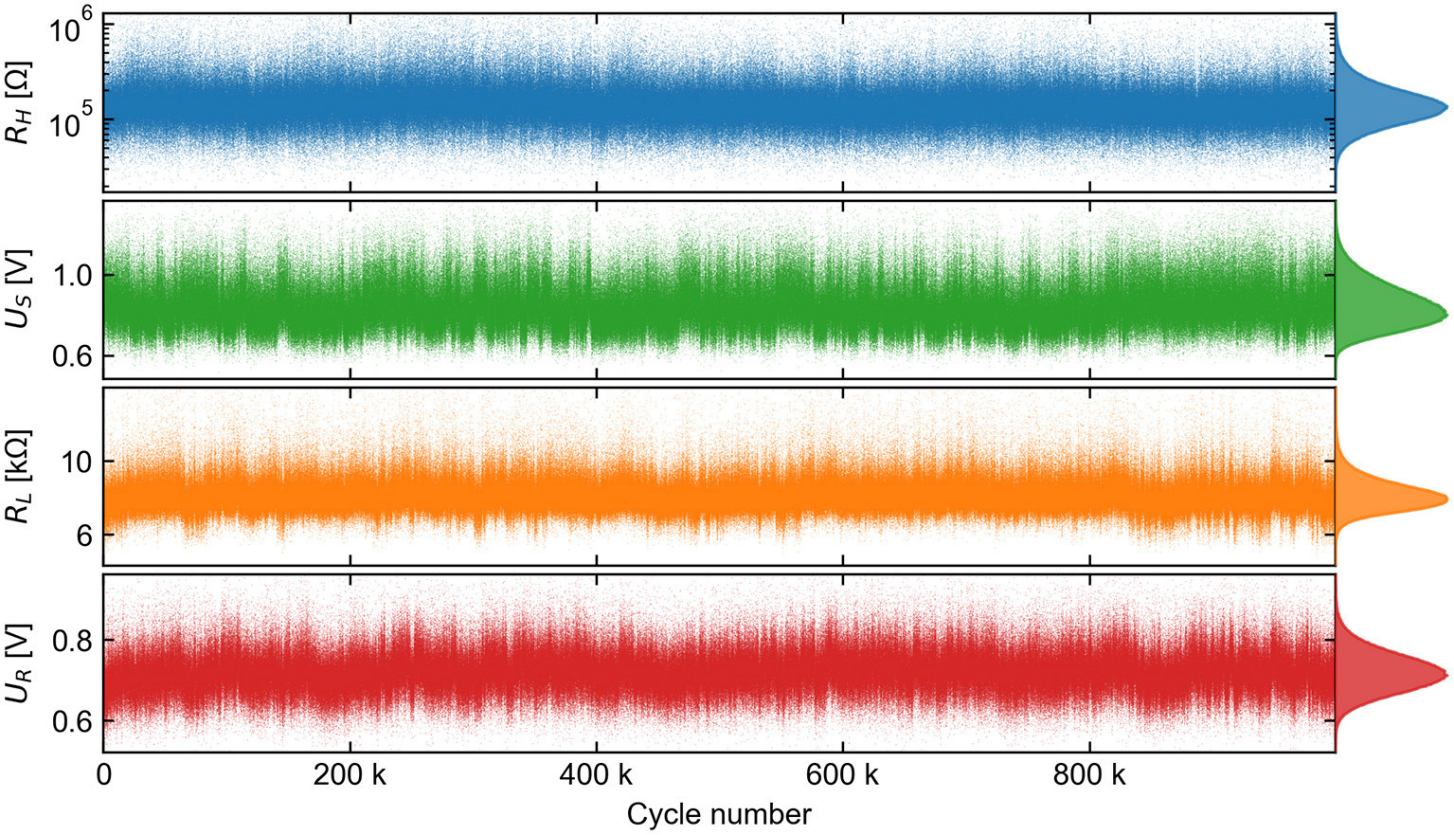
A view of the feature vector time series extracted from each of 10^6^ measured (*I, U*) cycles. Each feature, which represents either a resistance state or a switching voltage, has its marginal histogram shown on the right.

An overview of the result of this feature extraction is given in Figure 6. The 10^6^ cycles proceeded without significant long-term drift from the overall mean value,


(3) $$\begin{array}{l} {\bar{x}}_{n}=\left[\begin{array}{c} 166.5\;\text{k}\Omega \\ 0.85\;\text{V}\\ 8.2\;\text{k}\Omega \\ 0.72\;\text{V} \end{array}\right], \end{array}$$

but with significant variations in each feature between cycles. A prominent characteristic of this data is that it is strongly correlated over long cycle ranges, as quantified in **Figure 14**. The asymmetric marginal distributions for each of the features were very well resolved due to the large number of samples, and they did not accurately converge to any analytical probability density function (PDF) in common use, including the normal and log-normal.

### Stochastic modeling

**Figure 7:**
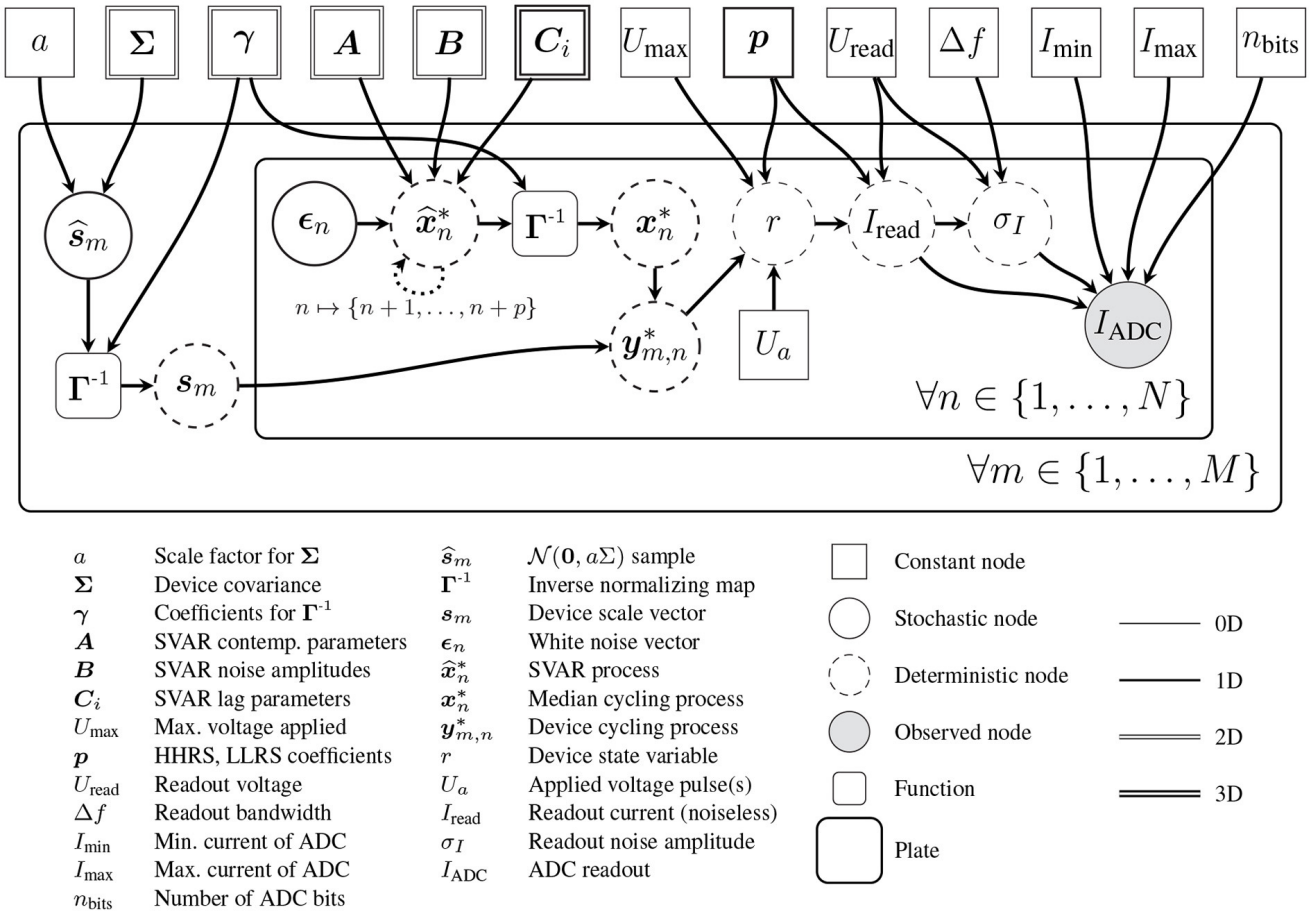
Graphical model depicting the relationships between all parameters and latent variables involved in the stochastic synapse model. Plate notation is used to represent *N* switching cycles of *M* devices, each yielding an observed readout current. The dotted recurrent arrow denotes a connection to each of the *p* following frames, as needed by the history dependent stochastic process.

This section will introduce the statistical methods used to model the internal states of an array of synaptic ReRAM devices, including CtC and DtD variability effects. The handling of voltages applied to the cells as well as the simulation of realistic readouts of the resistance states will also be established. To help orient the reader, the overall structure of the generative model that will be described is provided in advance in Figure 7.

#### Cycle-to-cycle variations

In seeking to represent the input time series *x*_*n*_ with a stochastic process, the main goals are to recreate the marginal distributions as well as the correlation structure of its vector components. To achieve the first goal with high generality, we use an approach based on transformation of the measured densities to and from the standard normal distribution N(0,1). This way, a single process can be used to achieve any set of marginals presented by the input data, with the relatively unrestrictive requirement that this base process generates normal marginals. Notationally, we define and apply an invertible, smooth mapping **Γ**:ℝ^4^ → ℝ^4^ that normalizes the marginal distributions of the vector components,


(4) $$\begin{array}{l} x_{n}={\left[\begin{array}{c} R_{H}\\ U_{S}\\ R_{L}\\ U_{R} \end{array}\right]}_{n}\overset{\Gamma }{\underset{\;}{\to }}{\left[\begin{array}{c} {\hat{R}}_{H}\\ {\hat{U}}_{S}\\ {\hat{R}}_{L}\\ {\hat{U}}_{R} \end{array}\right]}_{n}={\hat{x}}_{n}, \end{array}$$

where a hatted variable signifies that it is distributed as N(0,1). We then construct a base process ${\hat{x}}_{n}^{*}$ whose marginals are normal, and finally transform its output back to the original data distributions *via* the inverse map **Γ**^-1^. The overall process $x_{n}^{*}$ is thus defined,


(5) $$\begin{array}{l} {\hat{x}}_{n}^{*}={\left[\begin{array}{c} {\hat{R}}_{H}^{*}\\ {\hat{U}}_{S}^{*}\\ {\hat{R}}_{L}^{*}\\ {\hat{U}}_{R}^{*} \end{array}\right]}_{n}\underset{\to }{\Gamma^{-1}}{\left[\begin{array}{c} R_{H}^{*}\\ U_{S}^{*}\\ R_{L}^{*}\\ U_{R}^{*} \end{array}\right]}_{n}=x_{n}^{*}, \end{array}$$

where a star indicates a generated random variable to distinguish from variables originating from measurement data.

**Figure 8:**
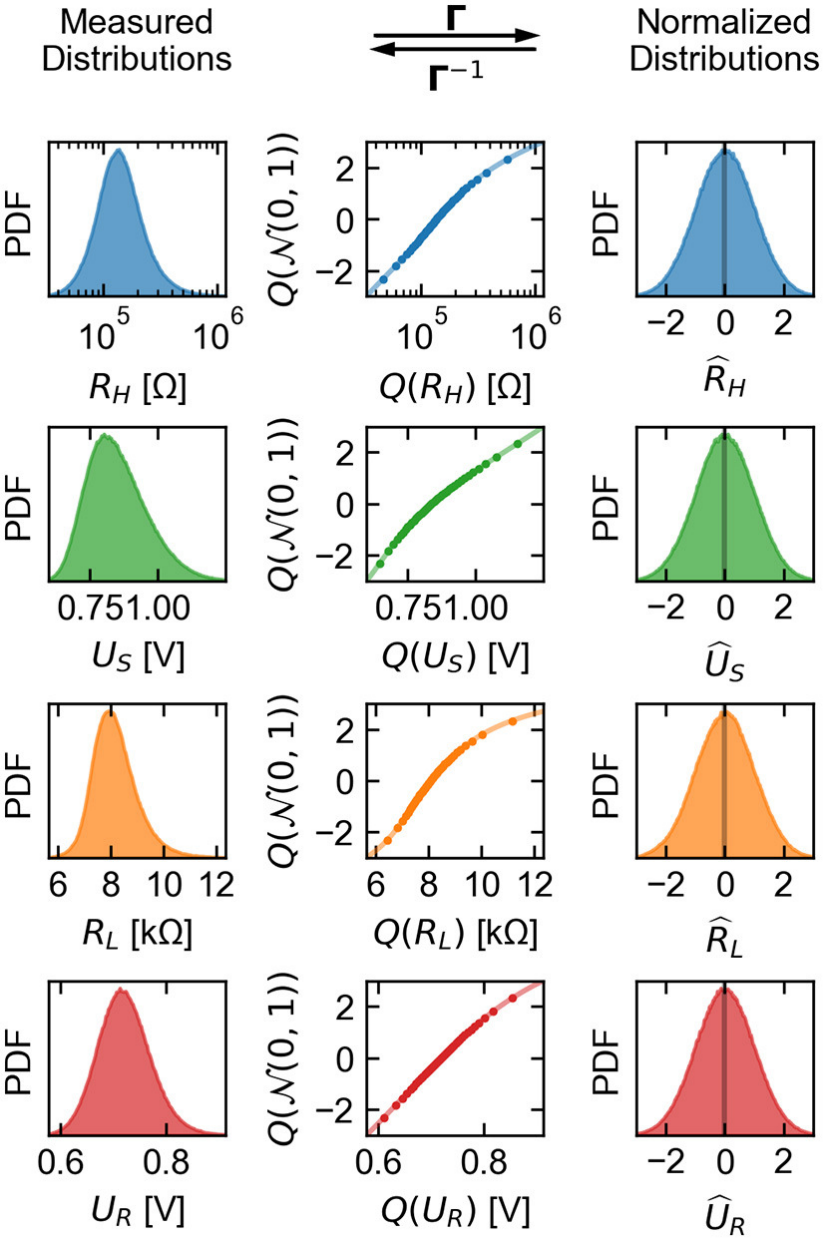
Visualization of the invertible normalizing transformation Γ that is applied to the measured feature vectors before fitting with a base stochastic process. The left column shows the marginal PDFs of the vector time series ***x***_*n*_ extracted from measurement. The center column shows the input and output quantile-quantile plots with the fitted log-polynomial function used to transform the distributions (here, *Q* denotes the quantile function of its argument). The right column is the result of applying **Γ** to the input data, producing ${\hat{x}}_{n}$ whose elements are normally distributed.

This type of density transformation procedure is a widely used technique for working with arbitrary distributions, which finds application in a variety of fields and can be constructed in many different ways (Cario and Nelson, 1996; Rezende and Mohamed, 2015). While the transformation is trivially constructed in the case where the target quantile function and its inverse are each analytically defined, we do not make this assumption in the present scenario. A simple numerical method in this case is a so-called quantile transform, where the input and output quantile functions are each discretely sampled and the transformation is defined through a direct map between bins or through interpolation. The main requirement for **Γ** in our model, however, is that its inverse (Equation 5) is easy to evaluate without causing cache misses due to memory access, thus it is preferable to avoid referencing and interpolation of large look-up tables. The forward transformation (Equation 4), on the other hand, only needs to be computed once for model fitting and is not used for the generating process. We therefore define **Γ**^-1^ as essentially a quantile transform, operating on each feature independently, that is evaluated from a fit of the quantiles to a specific analytic function. Namely,


(6) $$\begin{array}{l} \Gamma^{-1}({\hat{x}}_{n})=\exp\left[\begin{array}{c} \gamma_{1}({\hat{R}}_{H,n})\\ \gamma_{2}({\hat{U}}_{S,n})\\ \gamma_{3}({\hat{R}}_{L,n})\\ \gamma_{4}({\hat{U}}_{R,n}) \end{array}\right]=x_{n}, \end{array}$$

where γ_1_–γ_4_ are each 5th degree polynomials, and the exponential function is applied element-wise. The coefficients of the polynomials are fit to standard normal quantiles vs. those of the respective (log) features, sampled at 500 equally spaced values between 0.01 and 0.99. The fitted polynomials are checked for monotonicity within four standard deviations above and below zero, and the forward transformation,


(7) $$\begin{array}{l} \Gamma (x_{n})=\left[\begin{array}{c} \gamma_{1}^{-1}(\log R_{H,n})\\ \gamma_{2}^{-1}(\log U_{S,n})\\ \gamma_{3}^{-1}(\log R_{L,n})\\ \gamma_{4}^{-1}(\log U_{R,n}) \end{array}\right]=\;{\hat{x}}_{n}, \end{array}$$

is computed using numerical inverse of the **γ** polynomials. A visualization of the function Γ as well as the marginal histograms corresponding to input series ***x***_*n*_ and output series ${\hat{x}}_{n}$, are shown in Figure 8.

Now that we have transformed the input measurement data into a normalized vector time series ${\hat{x}}_{n}$, a suitable stochastic process will be chosen for fitting. This process should serve as a useful approximation to the true physical mechanisms that generated the data, capturing the long-range correlation structure of the observed features. Time series analysis is broadly used across scientific and engineering domains, but despite its applicability to the rich statistical behavior displayed by resistive switching devices, device models have not yet widely employed dependent stochastic processes. Many models and analyses assume for convenience that features are independently and identically distributed according to a normal or lognormal PDF (Chen, 2015; Li et al., 2017). However, there is not a strong theoretical basis for this assumption in a highly nonlinear and path-dependent system based on continuous evolution of conducting filaments. Dependent stochastic processes, on the other hand, more appropriately allow for a description of the dependence of future states on past states.

Simple models in the category of Markov chains have been considered as generating processes for memory cells. A rudimentary example is a 1-dimensional random walk process, where each future state is computed as a random additive perturbation on the previous state (Bengel et al., 2020). While random walk represents a reasonable short-range approximation, it has the well known property that the expected absolute distance between the initial value and the *N*th value is proportional to $\sqrt{N}$ for large *N*, causing the process to eventually drift to unphysical values without the use of artificial constraints.

Autoregressive (AR) models are simple univariate processes sharing some characteristics of random walk, but based additionally on a deterministic linear dependence on past observations. Each new term of an AR(*p*) (AR of order *p*) model is computed by linear combinations of *p* previous (lagged) values together with a noise term, producing processes that are wide-sense stationary and mean-reverting within suitable parameter ranges (Hamilton, 1994; Lütkepohl, 2005). The few times they have appeared in the literature, low order models like AR(1) and AR(2) were used to describe state variables independently (e.g., a sequence of high and/or low resistance states) (Fantini et al., 2015; Roldán et al., 2019). Here we pursue a more comprehensive statistical description of the interrelations between the different variables contained in the vectors ${\hat{x}}_{n}$ which takes into account long-range correlations *p* ≫ 1. This is enabled by using a VAR(*p*) model (vector AR of order *p*), which is the multivariate counterpart of the AR model applicable to discrete vector time series (Hamilton, 1994; Lütkepohl, 2005).

**Figure 9:**
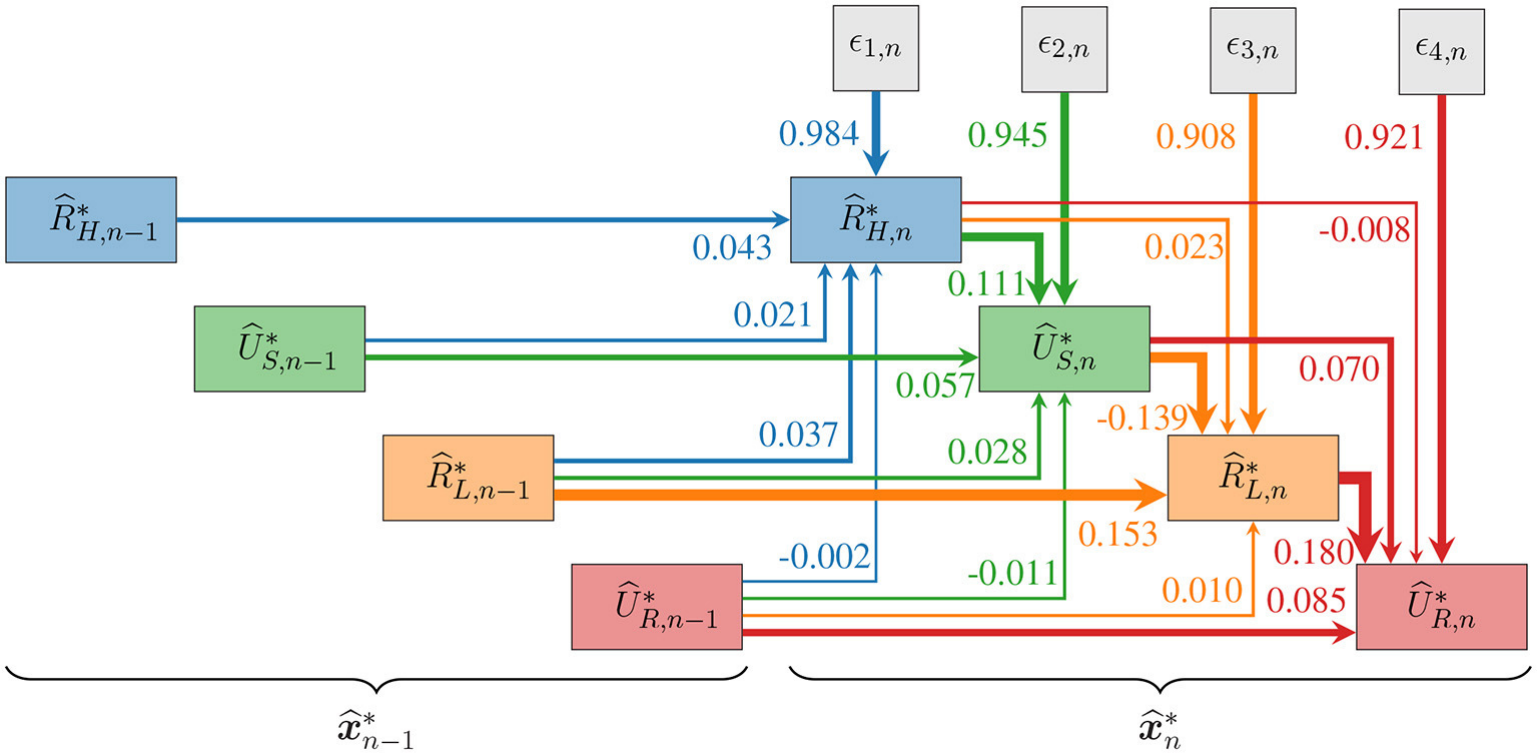
A weighted graph displaying the causal structure of the utilized SVAR(*p*) process, showing the nearest temporal contributions to realizations of the random vector ${\hat{x}}_{n}^{*}$. Arrow weights show the model parameters contained in ***A***, ***B*** and the upper triangular part of ***C***_1_ when fit with *p* = 100. The actual SVAR(*p*) model uses many more connections than shown (16*p*+10), so that each variable is impacted by all past values of all other variables within cycle range *p*.

We adopt in particular a Structural VAR (SVAR) formulation of the model, which is a factorization that makes the relationships between the contemporaneous (same index) variables explicit. The model has the form


(8) $$\begin{array}{l} A{\hat{x}}_{n}^{*}=\overset{p}{\underset{i=1}{\sum }}C_{i}{\hat{x}}_{n-i}^{*}+B\epsilon_{n}, \end{array}$$

where ***A***, ***B***, and ***C***_*i*_ are 4 × 4 matrices of model parameters, and ***ϵ***_*n*_ is a 4-dimensional standard white noise process. With this formulation we impose a general structure of causal ordering for the generated random variables consistent with the chronological chain of measurement events. Within this structure, each variable may have a causal and deterministic effect on all future variables within range *p*, as visualized by the graph of Figure 9. The size of these effects are all subject to fitting *via* the coefficients of the model. Constraints on the structural parameters,


(9) $$\begin{array}{l} A=\left[\begin{array}{cccc} 1 & 0 & 0 & 0\\ A_{21} & 1 & 0 & 0\\ A_{31} & A_{32} & 1 & 0\\ A_{41} & A_{42} & A_{43} & 1 \end{array}\right],B=\left[\begin{array}{cccc} B_{11} & 0 & 0 & 0\\ 0 & B_{22} & 0 & 0\\ 0 & 0 & B_{33} & 0\\ 0 & 0 & 0 & B_{44} \end{array}\right] \end{array}$$

enforce the desired causal structure while assuming an uncorrelated noise driving process. Model fitting was performed using the Python statsmodels package (Seabold and Perktold, 2010), wherein a VAR(*p*) model is first fit by ordinary least squares regression, and a maximum likelihood estimate is then used to determine the structural decomposition.

#### Device-to-device variations

So far, we have only considered the statistical modeling of the cycling process of a single memory cell. However, the purpose of the presented model is to simultaneously simulate a large number of cells in a network. Individual memory devices on a wafer generally show statistical variations, mainly arising due to defects and non-uniformities in fabrication (Fantini et al., 2013; Dalgaty et al., 2021). These DtD variations depend strongly on the lithography processes and materials used. They can also originate from intrinsic factors and are influenced by conditions during the electroforming of each cell (Butcher et al., 2012; Zhao et al., 2014). Because of the potential positive or negative impact on network performance, it is important for the model to account for the DtD variability (Moon et al., 2019; Dalgaty et al., 2021).

The electrical effect of device variability is modeled with each cell using a modification of the same underlying SVAR cycling process. Device-specific processes are defined as members of a parametric family of processes, all based on element-wise scaling of $x_{n}^{*}$, where the scaling factors are themselves random vectors. The specific process is denoted


(10) $$\begin{array}{l} y_{m,n}^{*}=s_{m}⊙x_{n}^{*}, \end{array}$$

where *m* = {1, 2, …, *M*} is the device index, ⊙ is the Hadamard (element-wise) product, and *s*_*m*_ are 4 × 1 random vectors drawn from a fixed distribution at cell initialization.

The distribution of ***s***_*m*_ is chosen so that the features of the median cycles of different devices are distributed and correlated in the same way as the measured cycling data ***x***_*n*_. This choice reflects that the covariations of switching features DtD arise in the same physical system with causes and effects that are comparable to those of the CtC variations. To this end, random vectors ${\hat{s}}_{m}$ are drawn from a multivariate normal (MVN) distribution and Γ-1 is then reused to map them to the measured CtC distribution,


(11) $$\begin{array}{l} s_{m}=\;\Gamma^{-1}({\hat{s}}_{m})⊘\;\Gamma^{-1}(0),\text{ where }{\hat{s}}_{m}\sim \mathbb{N}(0,a\;\Sigma ). \end{array}$$

Here, the denominator of the Hadamard division (⊘) sets the median scale vector to the identity, $\Sigma =\text{cov}\left({\hat{x}}_{n}\right)$ is the sample covariance of the normalized measurement data, and *a* is a free scalar parameter providing adaptability to different DtD covariance levels. A robust determination of *a* requires measurement of many switching cycles across a large number of devices of interest. Values in the range *a* ∈ [1, 1.5] approximately correspond to published DtD measurement samples (Fantini et al., 2013; Dalgaty et al., 2021), but improved processing and electroforming procedures may justify the use of *a* < 1.

#### Control logic

**Figure 10:**
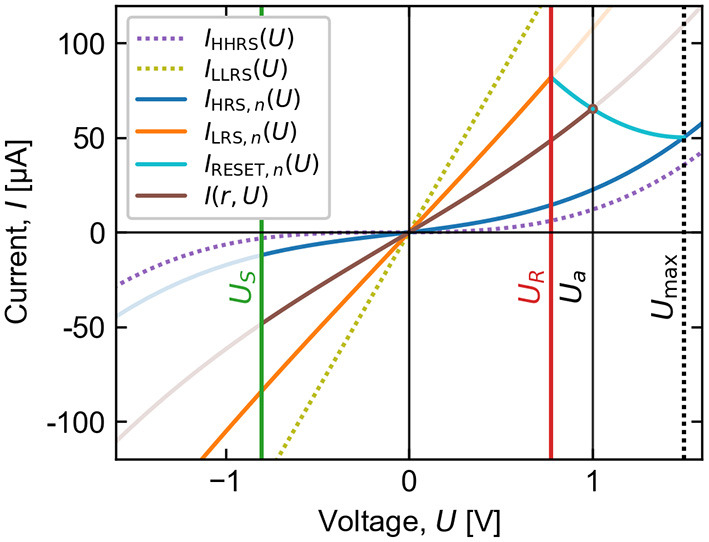
Conduction polynomials and threshold voltages allow reconstruction of (*I, U*) cycles from generated feature vectors. Simulated resistance switching is such that the conduction state *I*(*r, U*) induced by an applied voltage *U*_*a*_ intersects the reconstructed cycle at *U* = *U*_*a*_. For visual simplicity, the cycle shown begins and ends in the same HRS (*R*_*H, n*_ = *R*_*H, n*+1_).

As components of a network, each simulated cell possesses a resistance state that encodes the weight of a connection. Voltage pulses directly applied to the cells are used to produce resistance state transitions to update the weights. In this model, applied voltage pulses are distinguished only by a scalar amplitude *U*_*a*_, whether they are in fact square waveforms or they have a more complex shape of an action potential. Although ReRAMs are known to be highly time-dependent devices (Menzel et al., 2015), we assume here that the duration of the pulses are appropriately matched to the experimental timescale, such that a simulated voltage pulse of a given amplitude produces an effect comparable to the experimental voltage sweep at the instant it reaches that same amplitude. Possible state modifications in response to an input pulse is computed with respect to *I, U* sweeps that are reconstructed from each stochastic feature vector generated for each cycle as illustrated in Figure 10.

As previously specified in Equation (1), every possible electrical state of a device is assumed to correspond to a polynomial *I*(*U*) dependence parameterized by a state variable *r*. It is straightforward to calculate that the state variable for a curve passing through an arbitrary (*I, U*) point is uniquely given by the function


(12) $$\begin{array}{l} r\left(I,U\right)=\frac{I_{\text{LLRS}}\left(U\right)-I}{I_{\text{LLRS}}\left(U\right)-I_{\text{HHRS}}\left(U\right)}. \end{array}$$

Therefore, the state variable corresponding to any static resistance level *R* (evaluated at *U*_0_) can be calculated using


(13) $$\begin{array}{l} r(R)=\frac{I_{\text{LLRS}}(U_{0})-U_{0}R^{-1}}{I_{\text{LLRS}}(U_{0})-I_{\text{HHRS}}(U_{0})}. \end{array}$$

The *I*(*U*) curves for the electrical states corresponding to the HRS and LRS of each cycle, hereafter called *I*_HRS,n_(*U*) and *I*_LRS,n_(*U*), are defined according to equations (1) and (13) such that their static resistance equals the respective value of $R_{H,n}^{*}$ and $R_{L,n}^{*}$.

**Figure 11:**
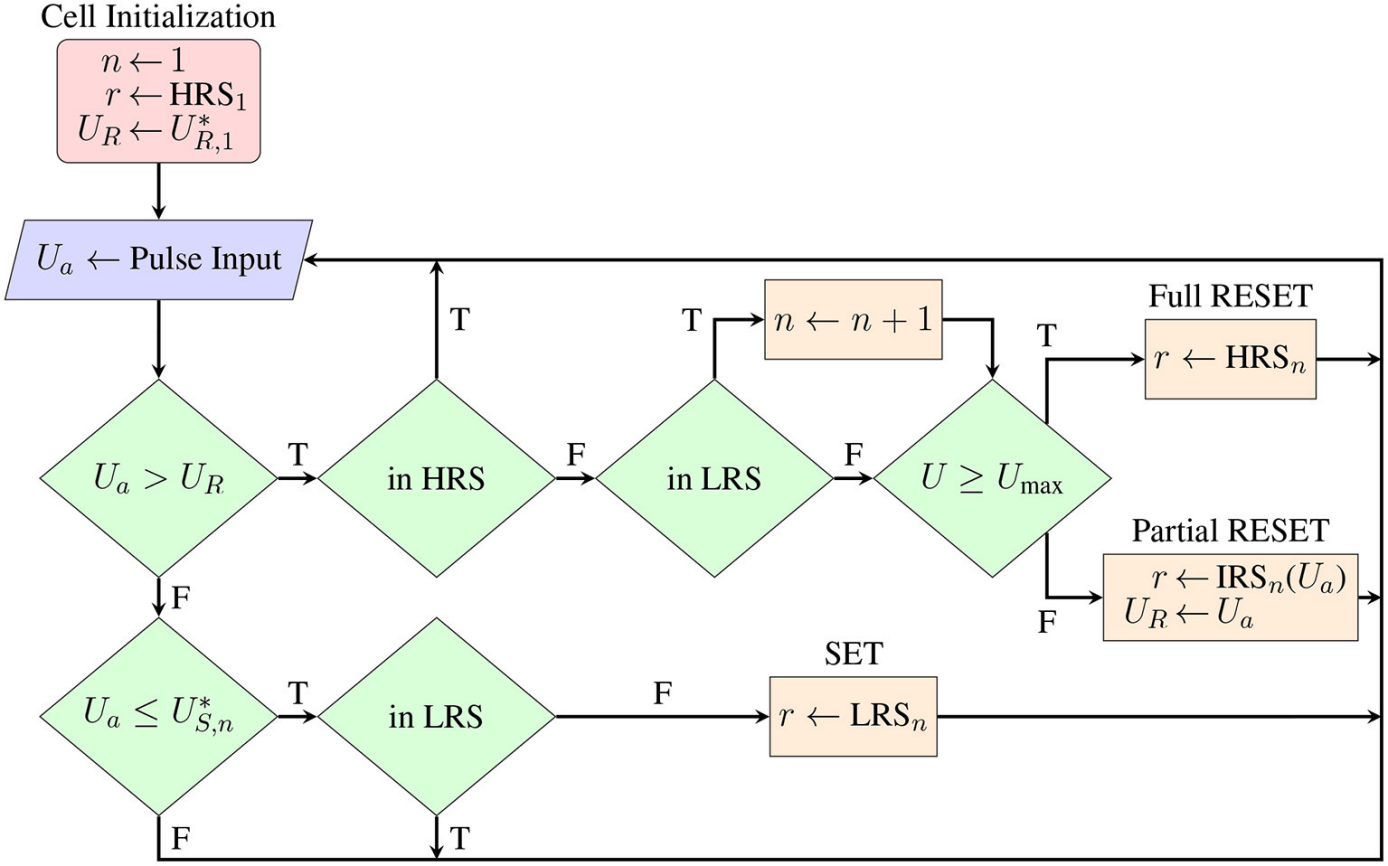
Logical flow chart showing how applied voltage pulses affect the state of each cell during simulation. Following the experimental observations, SET processes always occur abruptly below a threshold voltage, while partial switching is induced for a range of RESET voltages, with intermediate states bounded for cycle *n* by resistance values between *R*_*L,n*_ and *R*_*H,n*+1_. As resistance cycling progresses, later terms of the stochastic driving process are used for limiting resistance states and threshold voltages. Pulse amplitudes not producing a state change are efficiently disregarded.

Transitions between the HRS, LRS, and intermediate resistance states (IRS) in response to an applied pulse amplitude *U*_*a*_ follow an empirically motivated structure, represented by the flow chart of Figure 11. The SET transition for the *n*th cycle HRS_*n*_ → LRS_*n*_ may occur for negative voltage polarities and follows a simple threshold behavior, fully and instantaneously transitioning the first time a voltage pulse with amplitude $U_{a}\leq U_{S,n}^{*}$ is applied. In contrast, the RESET transition LRS_*n*_ → HRS_*n*+1_ occurs gradually in the positive polarity with increasing *U*_*a*_ in the range $U_{R,n}^{*}<U_{a}\leq U\text{max}$, where *U*max = 1.5 V is the maximum voltage applied in the voltage sweeping measurement. A transition curve *I*_RESET, n_(*U*) is defined to connect the (*I, U*) points of the two limiting states where the RESET transition begins and ends. The functional form of the transition curve is chosen to be the parabola with boundary conditions


(14) $$\begin{array}{lll} I_{\text{RESET,n}}\left(U_{R,n}^{*}\right) & = & I_{\text{LRS,n}}\left(U_{R,n}^{*}\right) \end{array}$$

(15) $$\begin{array}{lll} I_{\text{RESET,n}}(U_{\text{max}}) & = & I_{\text{HRS,n+1}}(U_{\text{max}}) \end{array}$$

(16) $$\begin{array}{lll} \frac{dI_{\text{RESET,n}}}{dU}{|}_{U=U_{\text{max}}} & = & 0. \end{array}$$

When a voltage pulse in the RESET range is applied, an IRS results which is calculated with reference to the transition curve such that *I*(*r,U*_*a*_) = *I*_RESET, n_(*U*_*a*_). Additional RESET pulses with larger amplitudes may be applied to incrementally increase the cell resistance, with HRS_*n*+1_ being reached only if *U*_*a*_≥*U*max, after which no further RESET switching is possible for the *n*th cycle. After either partial or full RESET, the resistance may only decrease again by entering the following LRS_*n*+1_ with a voltage pulse meeting the SET criterion $U_{a}\leq U_{S,n+1}^{*}$.

#### Readout

Simulated current measurements (readouts) for each individual cell can be generated given an arbitrary readout voltage input *U*_read_. The noise-free current level simply corresponds to evaluation of *I*(*r, U*_read_) for each cell. In any real system, however, current readouts are accompanied by measurement noise, which may impact system performance and even present a fundamental bottleneck. Furthermore, in digital systems current readouts are converted to finite resolution by analog to digital converters (ADCs). Due to constraints of power consumption and chip area, ADC resolution is often limited such that digitization is the dominant contributor to the total noise (Ma et al., 2019). Many additional noise sources can be considered, such as 1/*f* noise (Wiefels et al., 2020), but at minimum the Johnson-Nyquist noise and the shot noise should be included because they represent a lower bound of noise amplitude impacting all systems.

To account for measurement noise, each individual current readout includes an additive noise contribution drawn from a normal distribution. The noise amplitude is approximated from the Nyquist and Schottky formulas,


(17) $$\begin{array}{l} \sigma_{I}=\sqrt{\frac{4k_{B}TI_{\text{read}}\Delta f}{U_{\text{read}}}+2qI_{\text{read}}\Delta f}, \end{array}$$

where Δ*f* is the noise equivalent bandwidth, *k*_*B*_ is the Boltzmann constant, *T* = 300 K is the temperature, *q* is the electron charge, *I*_read_ is the noiseless current readout, and *U*_read_ is the voltage used for readout. The total current is then ideally digitized with an adjustable resolution *n*_bits_ between adjustable minimum *I*_min_ and maximum *I*_max_ current levels.

### Program implementation

To facilitate investigations of neuromorphic systems, model implementations designed to simulate arrays of devices were developed in the Julia programming language. Julia is a modern high-level language that is focused on performance and that provides an advanced ML and scientific computing ecosystem. Julia programs compile to efficient native code for many platforms *via* the LLVM compiler infrastructure, and a cursory analysis indicated that single threaded CPU performance of a Julia implementation is up to 5,000 times faster than a Python implementation. Furthermore, as modern computational resources are highly parallel, Julia's support for CPU multi-threading and GPU programming through CUDA.jl (Besard et al., 2019) is an important advantage.

All model parameters corresponding to the device characterized in this article, including different possible SVAR model orders, *p* ∈ [1, 200], are stored in a binary file which is read in by the program at startup. Each instantiated cell stores state information and *p* cycles of history using primarily 32-bit floating point numbers. The total memory footprint grows linearly with the chosen model order and is approximately 16*p*+56 bytes per cell. A reduced form VAR process is used to compute realizations of $x_{n}^{*}$, which are lazily evaluated along with the parabolic transition polynomials if and when they are needed. The majority of the necessary runtime computations are formulated as matrix multiplications, which are heavily optimized operations across many different contexts.

The present release contains two model implementations to suit a wide variety of computing platforms and use cases (Hennen, 2022). The first is a CPU optimized version wherein the cells of an array are individually addressable for read/write operations. These operations are naturally parallelized for multi-core processors by partitioning the cells and assigning each partition to independent threads of execution. The second implementation is a GPU accelerated version compatible with CUDA capable GPUs. This version uses a vectorized data structure and parallel array abstractions to take advantage of the implicit parallelism programming model of CUDA.jl. Here, all defined cells are always accessed simultaneously, with each read/write operation employing optimized linear algebra GPU kernels. While the GPU implementation integrates well with other ML components residing in GPU shared memory and achieves higher throughput per cell for large parallel operations, the CPU implementation obtains higher update rates for sparse operations commonly encountered in large-scale models (Pedroni et al., 2019, 2020).

## Results

**Figure 12:**
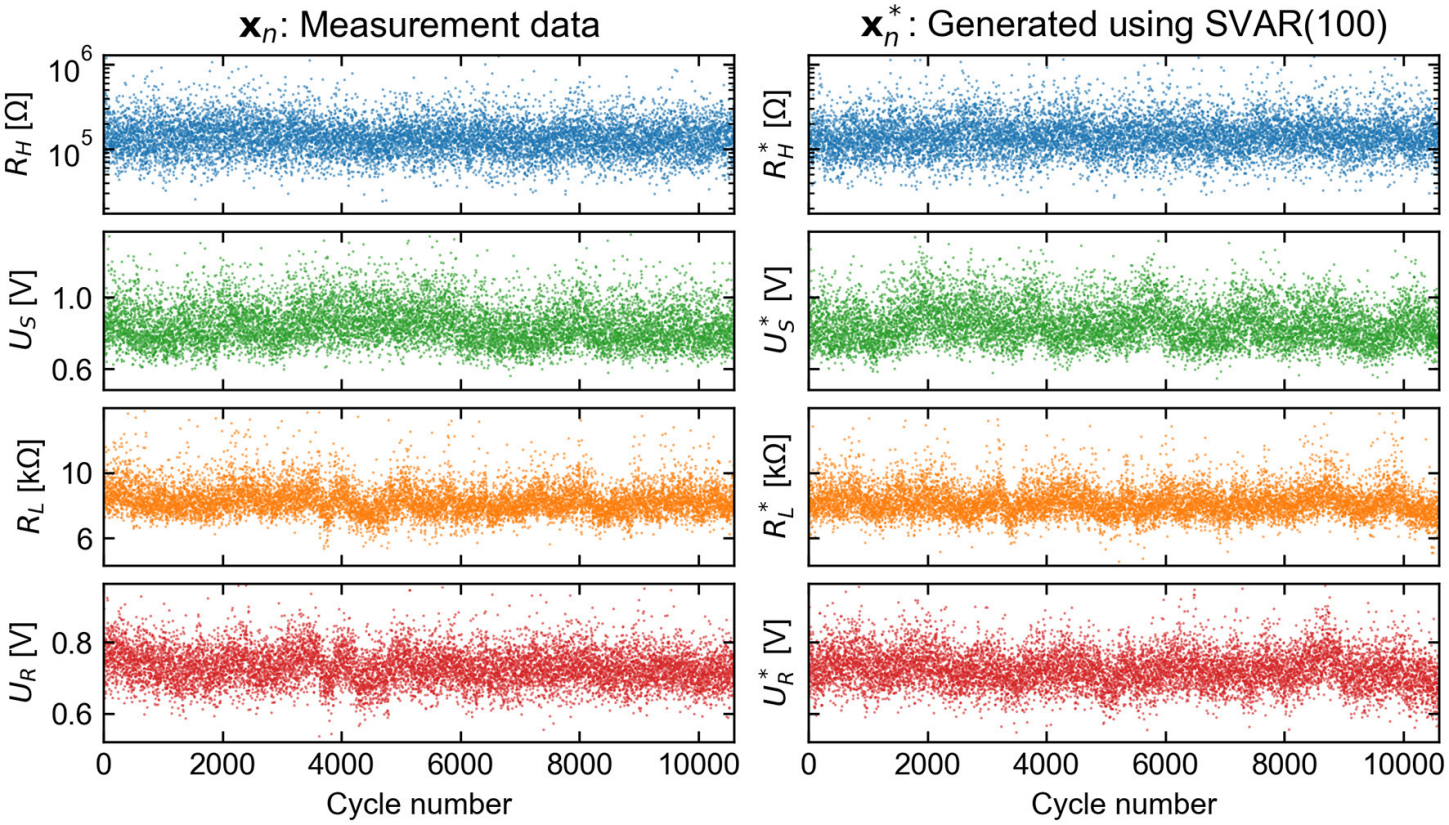
Comparison of feature time series extracted from measurement data and those generated by the SVAR-based model. The compared features converge to effectively equivalent distributions and the short-range behavior is qualitatively similar across thousands of cycles.

As shown visually in the scatterplot of Figure 12, the stochastic process $x_{n}^{*}$ generates data that closely resemble the measurement data ***x***_*n*_. The generated distributions match the empirical distributions so closely that it is difficult to visualize their difference. The Wasserstein metric is a distance function defined between probability distributions that can be used to quantify a small discrepancy (Kantorovich, 1960). The first Wasserstein distance was calculated element-wise and averaged across 100 realizations of $x_{n}^{*}$ with length 10^6^. The result,


(18) $$\begin{array}{l} {\bar{W}}_{1}(x_{n},x_{n}^{*})=\left[\begin{array}{c} 5,146\;\Omega \\ 937\;\text{μV}\\ 20\;\Omega \\ 356\;\text{μV} \end{array}\right], \end{array}$$

is much smaller than the mean feature vector, ${\bar{x}}_{n}$ (Equation 3), and independent of the chosen model order. This shows that the goal of reproducing the measurement distributions is well achieved for the input dataset by using the described method of probability density transformation.

**Figure 13:**
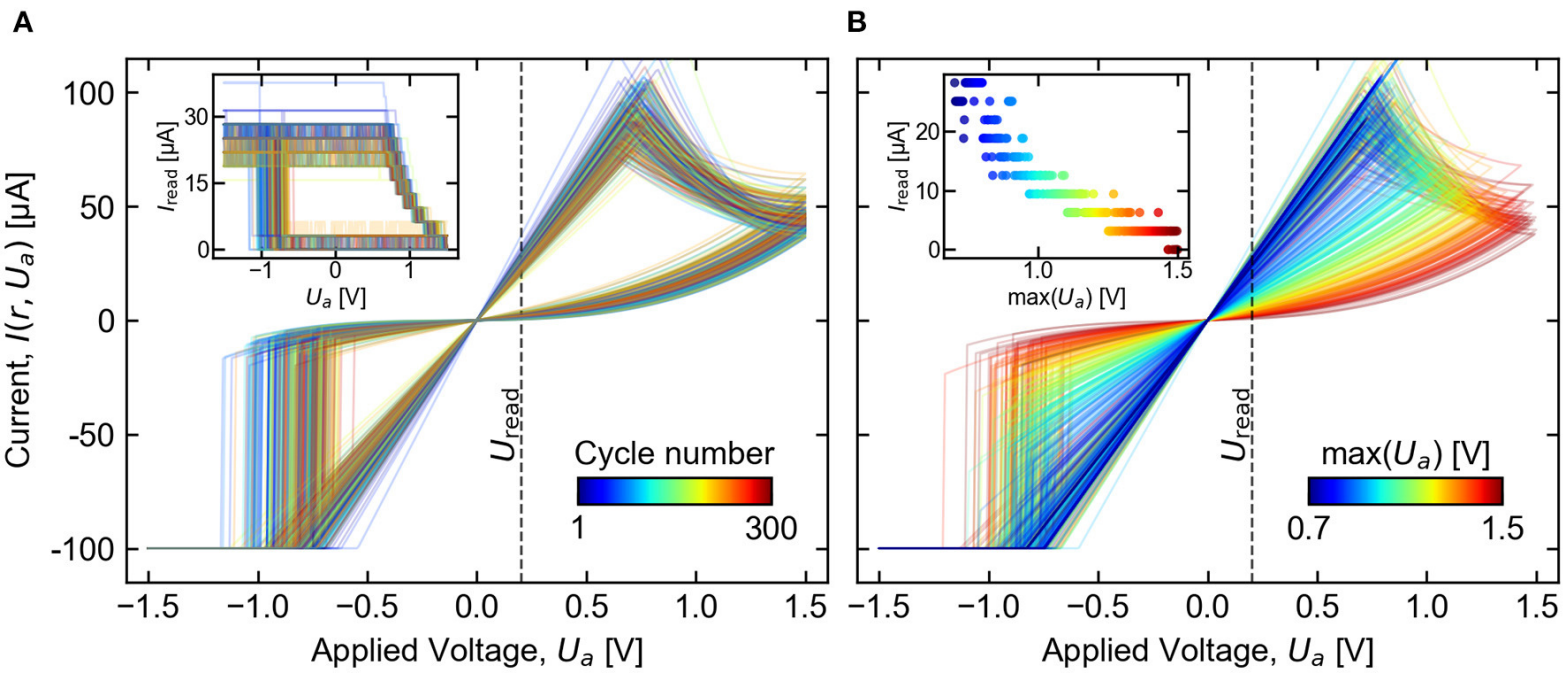
Two example simulations involving repeated cycling of a single device. Voltage pulse sequences were applied with varying amplitude following a triangular envelope, and the (*I, U*) characteristic of each cycle is plotted in a different color. Subplot **(A)** shows 300 consecutive cycles between the full voltage range ±1.5 V, with a readout performed after every pulse (inset). Subplot **(B)** demonstrates multilevel capability with 300 cycles between –1.5 V and maximum voltage that increases each cycle, from 0.7 V to 1.5 V. Readouts following each cycle are shown in the inset. In each case, readouts were simulated using a fixed *U*_read_= 200 mV, including noise and 4-bit quantization between *I*_min_ = 0 μA and *I*_max_ = 40 μA.

Simulations of full (*I, U*) cycling measurements (Figure 13A) show close similarity with the measurement data of Figure 5. Multi-resistance-level capability is also demonstrated by a similar simulation involving partial RESET operations by changing the maximum voltage applied (Figure 13B). The dependence of the resulting HRS value on the applied voltage reproduces a non-linear characteristic comparable to experimental findings (Park et al., 2013; Ambrogio et al., 2016).

**Figure 14:**
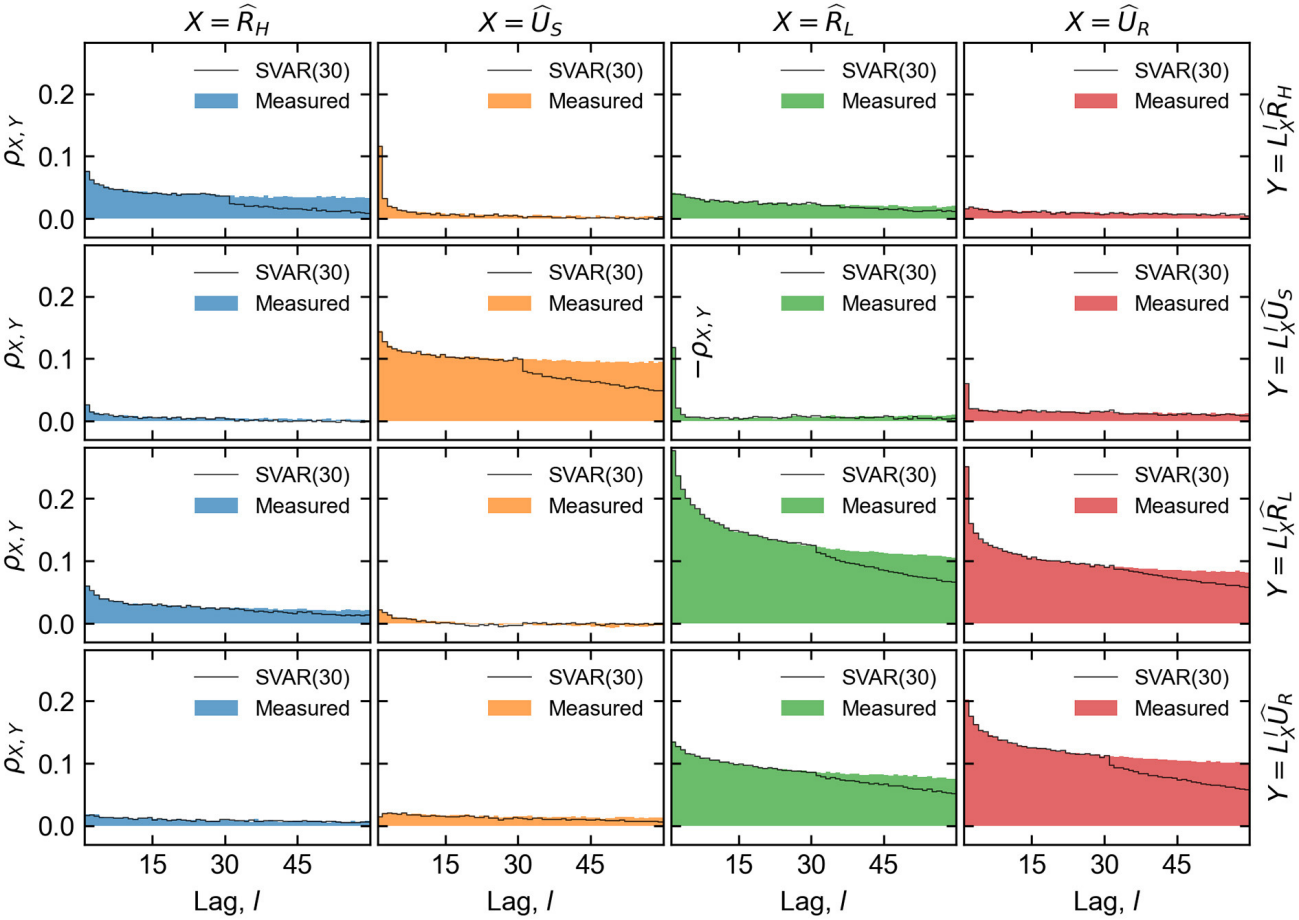
Auto- and cross-correlations of the normalized feature vector components, showing the Pearson coefficients ρ_*X,Y*_ of the variables specified in the subplot columns *X* and rows *Y* as a function of lag *l*. Row variables are lagged with respect to column variables, as denoted by the lag operators *L*_*X*_. A comparison between measurement data and data generated from SVAR(30) shows extremely close agreement up to cycle range 30. For lags larger than the chosen model order, some of the correlations of ${\hat{x}}^{*}$ decay more quickly than $\hat{x}$.

While a full structural analysis of the fitted SVAR(*p*) model parameters (***A, B, C***_*i*_) will not be presented here, a few aspects are worthy of note. For the fit corresponding to the particular device and measurement described in this work, the white noise terms are by far the dominant contributors to all four modeled features. The contemporaneous terms (***A***) and first order (***C***_1_) terms are the next most significant, which indicates that the most recent cell history is most relevant for generating the proceeding states. Nevertheless, input data correlations persist for many cycles, and the generating process $x_{n}^{*}$ successfully reproduces the overall correlation structure of the data up to at least *p* cycle lags, as shown in detail in Figure 14.

Although no physical effects were explicitly put into the model definition, it is important to recognize that the effects are quantitatively captured and put into a useful statistical context by the SVAR model fitting procedure. The model weights contained in ***A, B***, and ***C***_*i*_ quantify deterministic relationships between past and future variables even in the presence of large random fluctuations. As seen in the graph of Figure 9, the four strongest coefficients in the fitted model correspond to the relationships


(19) $$\begin{array}{ll} {\hat{R}}_{H,n}^{*} & \overset{0.111}{\underset{\;}{\to }}{\hat{U}}_{S,n}^{*}, \end{array}$$

(20) $$\begin{array}{ll} {\hat{U}}_{S,n}^{*} & \overset{-0.139}{\underset{\;}{\to }}{\hat{R}}_{L,n}^{*}, \end{array}$$

(21) $$\begin{array}{ll} {\hat{R}}_{L,n-1}^{*} & \overset{0.153}{\underset{\;}{\to }}{\hat{R}}_{L,n}^{*}, \end{array}$$

(22) $$\begin{array}{ll} {\hat{R}}_{L,n}^{*} & \overset{0.180}{\underset{\;}{\to }}{\hat{U}}_{R,n}^{*}, \end{array}$$

where weight of each relationship is printed above the arrows.

Comparable relationships between switching variables have been identified and discussed in physics-based models and simulations as well as in experimental studies involving various materials (Ielmini, 2011; Nardi et al., 2011, 2012; Nishi et al., 2015; Kim et al., 2016a,b; La Torre et al., 2016). According to relation 19, larger starting HRS values tend to contribute to a higher SET voltage, which is a well known effect due to a reduced driving force for ionic motion at a given applied voltage, as a larger HRS gives both reduced power dissipation as well as a reduced electric field in a thicker insulating gap. The subsequent LRS is strongly affected by the SET voltage (relation 20). This can be attributed to the runaway nature of the SET transition and a higher voltage initial condition, and is also connected with the dynamics of the current limiting circuitry (Hennen et al., 2021). The LRS value is also strongly correlated with the value of the previous LRS (relation 21), because of the influence of the residual filamentary structure from the previous cycle (Piccolboni et al., 2015). Lastly, relation 22 indicates that higher LRS values tend to have larger reset voltages, which has to do with a balance of factors influencing filament dissolution, including temperature and drift. This balance depends on the cell materials, operating regime, and internal series resistance (Ielmini et al., 2011).

### Benchmarks

As a benchmark of the throughput of write operations, repeated resistance cycling was induced on arrays of simulated cells under varying conditions. In each case, voltage pulse sequences to be applied to all defined cells were generated prior to the benchmarks, consisting of amplitudes ±1.5 V with alternating polarity. Defined as such, every pulse drives each cell through a transition into its next HRS or LRS. The read operation was benchmarked separately under equivalent conditions, reading out the entire array using a fixed readout voltage of *U*_read_ = 0.2*V*.

**Figure 15:**
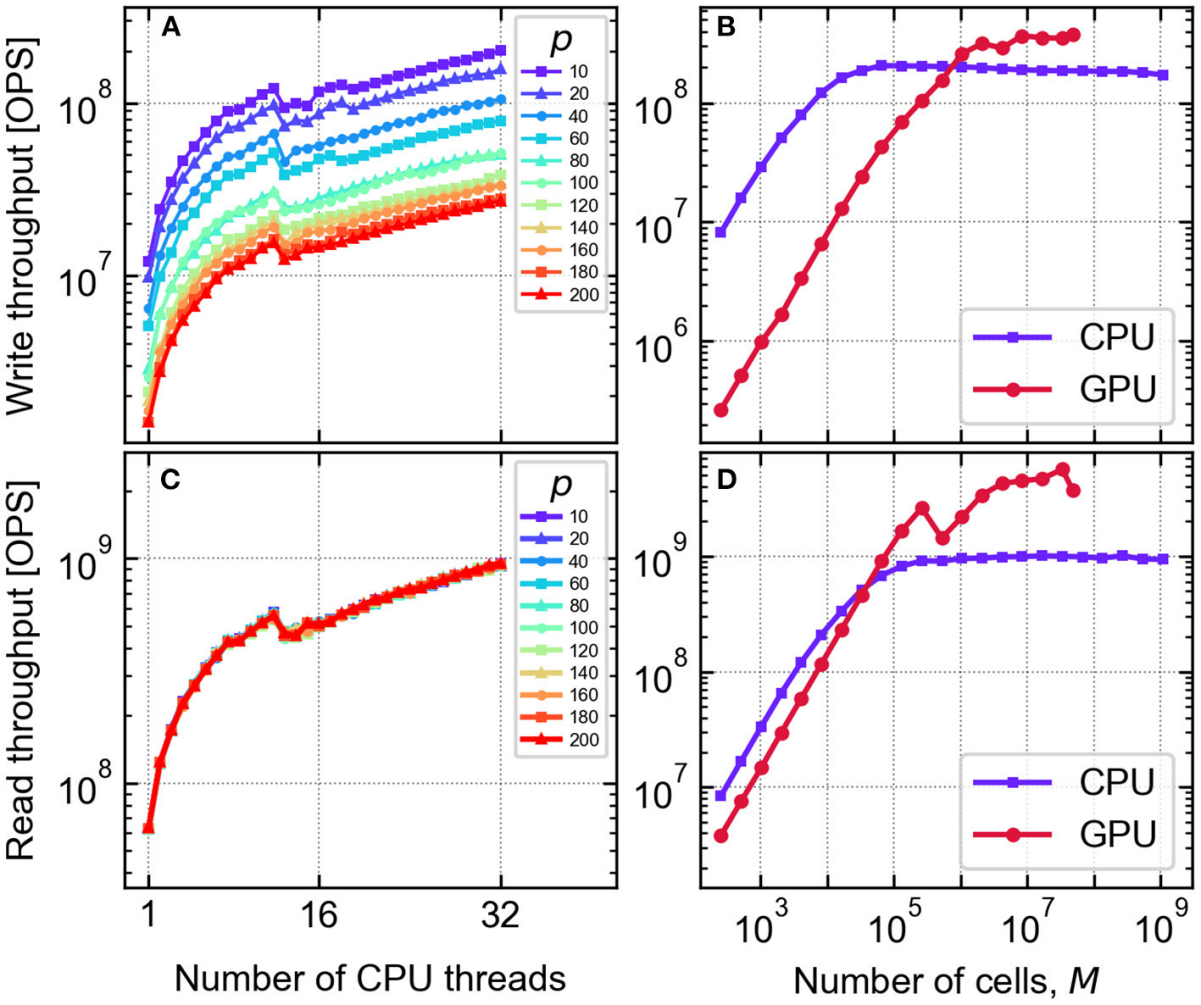
Benchmarks of the read/write operation throughput per cell of the Julia model implementations. In **(A,C)**, an array of 2^20^ (≈10^6^) cells are simulated on the CPU as a function of number of parallel threads spawned, and the VAR model order *p*. In **(B,D)**, the CPU (32 threads) and GPU implementations are benchmarked vs. the cell array size *M*, with *p* = 10.

The CPU benchmark was performed using an Intel Xeon Silver 4116 CPU, varying the cell array size *M*, the order of the VAR process *p*, as well as the number of threads used to perform the operations in parallel. The resulting read/write throughputs are summarized in Figure 15. Write throughputs up to 2 × 10^8^ operations per second (OPS) were obtained, which is equivalent to 5 ns per individual write operation. Read operations were nearly an order of magnitude faster than writes, with up to 10^9^ OPS or 1 ns per read operation. Due to the size of necessary matrix multiplications, increasing the VAR order *p* incurs a cost of write throughput, with a *p* = 100 model running approximately 4 × slower than one with *p* = 10. The read operation, in contrast, shows a negligible dependence on the VAR order.

The GPU accelerated version was benchmarked in an analogous way, using the same host machine with an NVIDIA TITAN RTX GPU device. The results are shown in dependence of the cell array size *M* in Figures 15B,D. The GPU implementation overtakes the CPU above *M* = 10^6^ parallel operations where the entire array is updated, and achieves 2 × faster updates and 5 × faster readouts for large arrays with *M* > 10^7^. However, CPU throughput is applicable to subsets of the array, and may retain an advantage for sparse operations.

## Discussion

In order to assess the potential of emerging synaptic devices, new lightweight and accurate device models are needed to constitute the millions/billions of weights used in modern machine learning (ML) models. Candidate memory cells such as ReRAM are highly non-linear stochastic devices with complex internal states and history dependence, all of which needs to be explicitly taken into account. In this article we introduced an efficient generative model for large synaptic arrays, which closely reproduces the statistical behavior of real devices.

Taking advantage of a recently developed electrical measurement technique (Hennen et al., 2021), we systematically fit the model to a dataset that is dense in relevant information about the device state evolution. Together with this new kind of measurement, our modeling approach helps complete a neuromorphic design feedback loop by defining a programmatic connection from the measured behavior of a fabricated device under the intended operating conditions directly to fitted model parameters. Probability density transformation of the underlying SVAR stochastic process gives the model the power to accurately reproduce nearly arbitrary distribution shapes and covariance structures across the switching cycles and across the separate devices. These features enable evaluation of network performance while automatically adapting to a wide variety of possible future device designs.

We provide parallelized implementations for both CPU and GPU, where up to 15 million cells per GB of available memory can be simulated at once. Benchmarks show throughputs above three hundred million weight updates per second, which exceeds the pixel rate of a 30 frames per second video stream at 4K resolution (3,840 × 2,160 pixels). Realistic current readouts including digitization and noise were also benchmarked, and are approximately an order of magnitude faster than weight updates. While speeds can be expected to improve with future optimizations, these benchmarks give a basis for estimating the scope of applicability of the model to ML tasks.

The implementation and the general concept of this model are naturally extendable. Although model parameters were adapted here to a specific HfO_2_-based ReRAM device, the method is applicable to a variety of other types of stochastic memory cells such as PCM, MRAM, etc. Four specific switching features were chosen in this demonstration to reconstruct (*I, U*) cycling behavior, but additional switching parameters can also be extracted from measurements and accommodated within this framework. Ideally informed by statistical measurement data, different functional forms, transition behaviors, time dependence, and underlying stochastic processes can each be substituted. Fitting may also be performed with respect to the output of physics-based simulations, thereby establishing an indirect link to physical parameters while achieving much higher computational speed. With these considerations, the model represents a flexible foundation for implementing large-scale neuromorphic simulations that incorporate realistic device behavior.

## Data availability statement

The original contributions presented in the study are included in the article/supplementary material, further inquiries can be directed to the corresponding author. A Julia implementation of the model is available on GitHub (https://github.com/thennen/StochasticSynapses.jl) and archived in Zenodo (https://doi.org/10.5281/zenodo.6535411).

## Author contributions

TH performed the data analysis, implemented the model, and wrote the manuscript. AE carried out the (*I, U*) measurement. JN and GM fabricated the ReRAM devices. RW and DW co-advised the project. DB conceived of the concept and was in charge of the project. All authors contributed to the article and approved the submitted version.

## Conflict of interest

Author GM is employed by Weebit Nano Ltd. The remaining authors declare that the research was conducted in the absence of any commercial or financial relationships that could be construed as a potential conflict of interest.

## Publisher's note

All claims expressed in this article are solely those of the authors and do not necessarily represent those of their affiliated organizations, or those of the publisher, the editors and the reviewers. Any product that may be evaluated in this article, or claim that may be made by its manufacturer, is not guaranteed or endorsed by the publisher.
